# COVID-19 and the *Infodemic*: An Overview of the Role and Impact of Social Media, the Evolution of Medical Knowledge, and Emerging Problems

**DOI:** 10.3390/healthcare10040732

**Published:** 2022-04-14

**Authors:** Francesca Corinti, Daniela Pontillo, Daniele Giansanti

**Affiliations:** 1 Facoltà di Medicina e Psicologia, Università Sapienza, 00185 Roma, Italy; fracorinti@hotmail.com (F.C.); daniela-pontillo@hotmail.com (D.P.); 2 Centro Tisp, Istituto Superiore di Sanità, 00161 Roma, Italy

**Keywords:** infodemic, pandemic, social media, COVID-19, infodemiology, infoveillance

## Abstract

The *infodemic* is an important component of the *cyber-risk* in regard to the poor and uncontrolled dissemination of information related to the COVID-19 pandemic. The purpose of this study was to perform a narrative review based on three points of view to allow for an overall picture of this issue. The points of view focused on: (a) the volume of use of social media (a key element of the infodemic) and the position of international health domain bodies; (b) the evolution of scientific production in the life sciences; (c) emerging issues. The research methodology was based on Google and PubMed searches and a qualification process based on a standard checklist and an evaluation of eligibility based on parameters with five score levels applied by two experts (plus one in case of discrepancy). The three points of view stressed the key role of social media as a dissemination tool of the *infodemic* among citizens. The impact on citizens depends on various social factors and involves indirect (e.g., vaccine avoidance) and direct risks such as mental problems and the risk of suicide. The widespread diffusion of social media, conveyed by mobile technologies, also suggests their use as countermeasures, calibrated based on citizens’ level of both technological and health literacy. Effective and promising countermeasures in this direction are based both on initiatives of contact by apps or SMS and the collection of data based on surveys and finalized to the particular intervention. The review also suggests as further areas of in-depth research: (a) to combat *high-level* *infodemic* produced by scientific publications that are not yet official (preprint) or that have undergone peer review with bias/distortion; (b) focusing on the impact of the *infodemic* considering its spread in different languages.

## 1. Introduction

Unlike other pandemics, COVID-19 exploded in an era in which new communication technologies based on social media have spread. Social media have peculiarities compared to other paper, television, and radio information dissemination systems. Their potential to disseminate information (true or false) places them at the center of attention of scholars and stakeholders in this delicate period due to the pandemic. Aspects such as the mechanism of dissemination of distorted information, the impact on the population, and the direct and indirect risks to public health are of continuous scientific interest today, and the pandemic represents, in a certain sense, an environment of growth regarding medical knowledge in this field.

### 1.1. New Communication Media Based on Social Media in the Pandemic Era

Social media are different from industrial media such as newspapers, television, and cinema. While social media are relatively low-cost tools that allow to publish and access information, traditional media require substantial financial investments to publish information [1,2]. Industrial media are commonly referred to as traditional, broadcasting, or mass media. A common feature of both social media and industrial media is the ability to obtain a large audience. Both a blog post and a television broadcast can reach millions of individuals [3]. There are different parameters [4,5] that are useful for both describing the difference between the two media and what comprises their different roles in spreading the information:

- Catchment area: both social media and industrial media offer everyone the opportunity to reach almost always a wide population.
- Accessibility: the means of production of industrial media are generally managedby companies or by the State. Everyone can access to social media and, near always, are free of charge.
- Usability: social media compared to industrial media does not require specific skills.
- Speed: the time to produce information by industrial media can be very long when compared to the time taken by social media.
- Permanence: The information produced by the social media can be modified/changed almost instantly. The information produced by industrial media are near always unchangeable.

The current pandemic is characterized by the presence of social media that can instantly transfer information to anyone with a smartphone. Therefore, it is essential to ensure that the information traveling through these systems is not distorted or misleading. Furthermore, the pandemic has been a driving force in the growth of mobile technologies, internet technologies in general, and social media. The number of users of mobile technologies and apps related to social media has grown considerably. In Italy, the data are reported in CENSIS (an Italian socioeconomic research institute) reports.

The latest Italian CENSIS report [6] outlined, in brief, the following: between 2019 and 2021, there was a strong increase in the use of the Internet by Italians (83.5% of users, with a positive difference of 4.2 percentage points), while those who used smartphones rose to 83.3% (a record growth compared to 2019: +7.6%) as well as a total of 76.6% who used social networks (+6.7%).

For print media, on the other hand, the now historic crisis is accentuated, starting with newspapers sold on newsstands, which in 2007 were read by 67.0% of Italians, reduced to 29.1% in 2021 (−8.2% compared to 2019). The same is true for weeklies (−6.5% in the two-year period) and monthlies (−7.8%), which were hard hit by the effects of the pandemic.

Among young people (14–29 years old), there was a further step forward in the use of media, in general, and of online platforms, in particular: 92.3% used WhatsApp; 82.7% YouTube; 76.5% Instagram; 65.7% Facebook; 53.5% Amazon; 41.8% video conferencing platforms (compared to 23.4% of the total population), 36.8% Spotify; 34.5% TikTok; 32.9% Telegram; 24.2% Twitter.

Even among the elderly (65 years and over), something is moving, given that internet use increased significantly from 42.0% to 51.4% and social media users increased from 36.5% to 47.7%. The need to maintain contact, at least virtually, with loved ones during the period of the most rigid isolation must have played a significant role in the confidence acquired in using the network by those over 65 years old.

These data, even if related to a country (Italy), suggest that attention to the correct dissemination of information must be directed particularly towards social media. These systems can rapidly disseminate both correct and distorted information. In the case of the spread of distorted information, they are sources of the *infodemic*.

### 1.2. Basis and Purpose the Review

Social media, using mobile technology, have been and still are an important component regarding the poor and uncontrolled dissemination of information related to the COVID-19 pandemic. It is important to understand: (a) how the *infodemic* evolved over time, also due to the evolution of social media and understanding how the latter occurred; (b) what are the positions of national and supranational bodies on this issue and the volume of the problem; (c) which themes are most dealt with; (d) what directions comprise future prospects.

In line with the above, we had the idea to propose a *narrative review* focused on the on the *infodemic* with the aim:

- To consider the dimensions of the problem and the positions of the most important national and international bodies in the health domain to answer the *key question 1*: “*what are* *the dimensions of the problem and the visions of the most important national and international bodies on the issue of the infodemic?*”
- To analyze the literature, since the definition of “*infodemiology*” and its actions do not yet a have consensus among international experts in order to answer to *key question 2:* “*how has the scientific literature evolved in this area? Is there is a movement towards scientific productions dedicated to the integration of consent?*”
- To analyze the themes emerging on the *infodemic* in the literature in order to answer *key question 3:* “*what are the issues addressed by the scientific community in this area?*”

This study was arranged according to following structure (also including Section 1 (Introduction), Section 4 (Discussion), and Section 5 (Conclusion)):

- Section 2 details the methodology of this review for each of the three goals;
- Section 3.1 is dedicated to the outcome of this review in terms of answering key question 1, reporting: (a) the volume of the problem worldwide in terms of the use of social media; (b) the position of the WHO, CDC, and EUC and their joint positions on specific joint initiatives with other bodies of international importance (i.e., WHO, UN, UNICEF, UNDP, UNESCO, UNAIDS, ITU, UN Global Pulse, and IFRC);
- Section 3.2 contains the output from the application of key question 2, reporting on the scientific production in this area, starting from the definition of the disciplines that revolve around the *infodemic* (i.e., *infodemiology* and *infoveillance*) up to a definition of the first important initiative towards an international consensus, including a recently produced a document with recommendations;
- Section 3.3 contains the output from the application of key question 3, reporting on the principal themes emerging in this field that were discovered during the literature review.

## 2. Methods

According to the purpose this narrative review, three points of view were focused on to allow for an overall picture of the issue. The methods have both a general and a specific framework. The general framework is that we followed a narrative checklist [7] as a supporting tool in the data synthesis. The specific framework for each goal was as follows:

First goal: Google searches were applied to (a) find international documents on the application of social media; (b) find documents on the positions of government agencies sorted by priority of importance. Two experts (plus an expert adjudicator in cases of discordance) performed these searches.

Second goal: Analyses were conducted on both the volume of PubMed’s scientific production on this topic and any relevant articles on (a) important passages related to the foundation of *infodemic* (before the pandemic) as a subject of study and (b) on events/current initiatives to integrate consent on actions to be carried out. The selection processes were carried out by the three experts (i.e., two experts plus an adjudicator) based on a qualification procedure as illustrated in the next point. Box 1 lists the search keys.

Box 1 **Search keys used (also with plurals).**

**Applied Keys**

*
Infodemic (Title/Abstract)
*

*
Infodemic (All Keys)
*

*
Infodemic (Title/Abstract)
*

*
Infodemic (Title/Abstract) AND (Social Media)
*

*
Infodemic (Title/Abstract) AND (Vaccine)
*

*
Infodemic (Title/Abstract) AND (Social Media) AND (Vaccine)
*

*Third goal:* The search was performed using PubMed. A specific qualification process was based on a scoring system (with different parameters and a score with 5 levels) applied by two experts (plus one adjudicator in cases of discordance) to include each reference (Table 1). It was possible to assign a score to these parameters ranging from a minimum score of one up to a maximum of five. As far as the “added contributions to the field” parameter was concerned, we used a weighting procedure. In consideration of the criticality of the first moments of the pandemic and relativizing the importance of the first period of the pandemic, the vote assigned was multiplied by:

- A factor × 1.3 (for studies published in the first three months of the pandemic);
- A factor × 1.15 (for studies published in the period ranging from three up to six months from the start of the pandemic).

A study was excluded if, regardless of the score, there were critical issues of conflict of interest (for example, it was conducted without guarantees of objectivity by the system manufacturer).

A study was included in the review if all parameters, after the weighting procedure, showed a score ≥ 3.

## 3. The *Infodemic*: Volume of the Problem and Position of the International Bodies, Scientific Production, and Key Points

### 3.1. The Infodemic: The Volume of the Problem and the Positions of Key International Bodies

#### 3.1.1. The Volume of the Problem

Various documents and sources were analyzed with reference to the volume of use of social media. Based on the list of priorities drawn up, the Statista source was proposed (https://www.statista.com, accessed on 11 April 2022) [8]. This source draws insights into consumers from industries and markets worldwide. Covering the offline and online world, the Statista Global Consumer Survey offers a global perspective on consumption and media usage.

The results of a search (data refer to October 2021) are shown in Table 2. This table shows the most used social media together with the type of interaction/service provided, manufacturer, and country of the manufacturer.

The results of another search (data refer to January 2022) are shown in Table 3.

This table highpoints the global diffusion of social media while also considering the technology used. Most users accessed social media through mobile technology, which is easy, wearable, and low cost, and *96.1%* of those with mobile internet accessed social media; *98.81%* of those who accessed social media did so with mobile internet. The outcome of both of the searches highlights a very large volume of social media use, confirming, at an international level, what has been pointed out in Italy at the national level [6] and, thus, corroborating the disruptive impact on the population of the dissemination of information (correct or distorted).

#### 3.1.2. The Position of the Key International Bodies

National and international bodies have dealt with the *infodemic* with reference to the role of social media. Here, there are some important positions of the “abovementioned government” entities in line with the *first objective of this study.* In the following are the positions selected by experts from key international bodies (also as joint positions) and from the US.

The WHO, whose primary role is to direct international health within the United Nations’ system and to lead partners in global health responses defines the *infodemic* as follows (literal quote) [9]:

“*An infodemic is too much information including false or misleading information in digital and physical environments during a disease outbreak. It causes confusion and risk-taking behaviours that can harm health. It also leads to mistrust in health authorities and undermines the public health response. An infodemic can intensify or lengthen outbreaks when people are unsure about what they need to do to protect their health and the health of people around them. With growing digitization—an expansion of social media and internet use—information can spread more rapidly. This can help to more quickly fill information voids but can also amplify harmful messages. Infodemic management is the systematic use of risk- and evidence-based analysis and approaches to manage the infodemic and reduce its impact on health behaviours during health emergencies. Infodemic management aims to enable good health practices through 4 types of activities:*

- *Listening to community concerns and questions*
- *Promoting understanding of risk and health expert advice*
- *Building resilience to misinformation*
- *Engaging and empowering communities to take positive action*”

According to the definition of *infodemic* by the WHO, the importance of the roles of digital technology and *social media* in this field appear.

In relation to the term *infodemic*, it is essential also to recall the important joint statement by nine international bodies: WHO, UN, UNICEF, UNDP, UNESCO, UNAIDS, ITU, UN Global Pulse, and IFRC [10]. We report the following passage (literal quote), particularly centered on *social media*:

“*We further call on all other stakeholders—including the media and social media platforms through which mis- and disinformation are disseminated, researchers and technologists who can design and build effective strategies and tools to respond to the infodemic, civil society leaders and influencers—to collaborate with the UN system, with Member States and with each other, and to further strengthen their actions to disseminate accurate information and prevent the spread of* …”

Always in relation to the term *infodemic*, it is essential to recall the European position, highlighted in a *speech by Vice President Věra Jourová at the EU Commission on 4 June 2020.* We report a brief section (literal quote) that particularly focuses on *social media* [11]:

“*The Coronavirus pandemic has been accompanied by an unprecedented ‘infodemic’, according to the World Health Organisation……*

*While this argument is happening in the US, Twitter, Facebook and other platforms are global and relevant for politicians and users in Europe as much as they are in the US. I have been saying for a long time that I want platforms to become more responsible, therefore I support Twitter’s action to implement transparent and consistent moderation policy. This is not about censorship. Everyone can still see the tweets. But it is about having some limits and taking some responsibility of what is happening in the digital world.*”

*In the US, the* Centers for Disease Control and Prevention also face this issue [12]. Among the recommendations related to deployment processes correlated to COVID-19 we found (literal quote): “*Prevent “infodemics”(an excessive amount of information about an issue that makes it difficult to identify a solution)—this builds trust, increases probability that health advice will be followed, and manages rumors/misunderstandings*.”

### 3.2. The Infodemic: The Evolution of Scientific Production

In line with the second objective of the study, this section deals with the evolution of scientific production in this area starting from the definition of the disciplines around the *infodemic* up to international consensus initiatives. We carried out incremental searches on *PubMed*.

These searches refer to the date 1 February 2022.

A first search was performed with the key *Infodemic (Title/Abstract)* [13], and 405 studies (34 reviews) were found, all of which were concentrated between 2020 and the reference date and were associated with the COVID-19 pandemic.

A free search with the key *Infodemic* [14] led to 407 papers, with 1 paper published in the year 2009 [15], which highlights how this term did not firsts appear scientifically with the COVID-19 pandemic but in an earlier era.

The first appearance of the term *infodemic* appeared in a study [15] proposed in an editorial by Eysenbach et al.

The authors also focused on the role of the *infodemiology* [16], the science that deals with *infodemic*. The term *infodemiology* was defined here [16] as a portmanteau of information and epidemiology. Although, in 2009, the spread of smartphones and *social media* had only just begun, the authors identified some basic concepts that can be extrapolated to date. These concepts concern, in particular, the definition and positioning of *infodemiology* and *infoveillance*. *Infodemiology* was defined (literal quote) [16] “*as the science of distribution and determinants of information in an electronic medium, specifically the Internet, or in a population, with the ultimate aim to inform public health and public policy*.” *Infoveillance* has been defined as the science that deals with surveillance in *infodemiology* [16].

The same authors of the study, the first to frame the science of *infodemiology*, applied the tools described above in the case of flu in 2006 [17] and highlighted how *infodemiology* is important for dealing with *cyber-behavior* [18]. The great explosion in this area, as we have seen, has occurred over the past two years associated with the pandemic. It covered both the pandemic in general [13] and (more recently) vaccines as well. The *infodemic* associated with the pandemic, in general, can lead to extreme behavior of underestimating the pandemic or terrorism. *Infodemic* associated with vaccines can cause avoidance [19].

A refinement of the search with the key *Infodemic* (*Title/Abstract) AND (Vaccine)* reports 64 papers [20] (15.8% of the total).

When we focused on the role of *social media* and searched with the key *Infodemic (Title/Abstract) AND (Social Media),* we found 223 papers [21] (56% of the total).

Further refining the research to highlight works dealing with vaccines, with the key *(Title/Abstract) AND (Social Media) AND (Vaccine),* we found 35 papers [22] (8.6% of the total).

There were 18 reviews in relation to the role of *social media* in the *infodemic* [23]; only 1 of these review also concerned vaccines [24].

A focus has also emerged among this research [25]. This study is a summary document produced by international experts after a meeting at the *First WHO Infodemiology Conference* [25].

The document is, in fact, an act of global orientation to address the *infodemic* with particular emphasis on the role of *social media*. The document, available since 15 September 2021, was developed through an analysis of the evidence, a review, and consensus of actions by the experts. In brief, it recommends:

- Evaluation and continuous monitoring of the effect of the infodemic in emergency periods;
- Detection of the signs of the spread of the phenomenon and the consequent risk;
- Implementation of mitigation actions of the phenomenon;
- Evaluation of intervention actions against the phenomenon and of the degree of resilience;
- Promotion of targeted interventions through the Internet.

### 3.3. The Infodemic: The Key Emerging Issues

In line with *the third objective* of this study, this section analyzes the issues on the *infodemic* by theme, based on the selected papers using both the proposed keys and the qualification process [26,27,28,29,30,31,32,33,34,35,36,37,38,39,40,41,42,43,44,45,46,47,48,49]. The papers were, as expected, mainly reviews; however, full scientific articles were also selected dealing with very peculiar aspects. The structure for this section starts with considering the methods of dissemination (a); then, it analyzes the social impact (b), the direct and indirect health risks (c), and the countermeasures (d).

#### 3.3.1. Methods of the Dissemination of the Infodemic

An *infodemic* has a source. This source may be a *misinformation* or a *disinformation*. It is important to clarify the differences between these two terms. Indeed, they have different roles in the *infodemic*. The authors of [26] make a differentiation based on what they call “the cognitive domain”. Thus, it may be said that “disinformation” refers to the deliberate creation or sharing of false information, whereas “misinformation” is not intended to mislead the receiver.

As for *disinformation*, in the Cambridge dictionary we find [27]: “*false information spread in order to deceive people. e.g., they claimed there was an official disinformation campaign by the government*.”

As for *misinformation*, in the Cambridge dictionary we find [28]: “*wrong information, or the fact that people are misinformed. e.g., there’s a lot of misinformation about the disease that needs.”*

There are several methods, now recognized, for the dissemination of the *infodemic*, whether it is *misinformation* or *disinformation*. A recent systematic review [29] conducted a literature search covering 12 scholarly databases to retrieve various types of peer-reviewed articles that reported the causes [29].

Social media usage, low level of health/eHealth literacy, and fast publication process and preprint service are identified as the major causes of the Infodemic.

In addition, the vicious circle of human rumor-spreading behavior and the psychological issues from the public (e.g., anxiety, distress, and fear) emerge as characteristics of the *infodemic*.

*Three important concepts* emerged, corroborated also by other reviews.

The *first concept* relates to the importance of social media as a key factor for the *infodemic*, both as a vehicle of communication and as regards the mastery of correct use (literacy). This was also confirmed in another review [30] that stressed the magnitude of the problem of COVID-19 misinformation on social media, its devastating effects, and its intricate relation to *digital health literacy*. As it was highlighted in [44], digital health literacy can help improve prevention and adherence to a healthy lifestyle, improve capacity building, and enable users to take the best advantage of the options available, thus strengthening the patient’s involvement in health decisions and empowerment and, finally, improving health outcomes.

The *second concept* is that there is also a *high-level infodemic* (HLI) generated, for example, from the scientific literature of the preprints—scientific works published before the peer review process. The presence of this HLI was also highlighted in [31] and extended to official academic and institutional publications of any type. The authors highlighted this among the causes of the exponential increase in COVID-19-related publications that often included biases in the peer-review and editorial process.

The *third concept* is the important role of word of mouth with the *infodemic,* which follows a peer-to-peer mechanism.

#### 3.3.2. The Social Impact

A recent scoping review [32] that focused on the social impacts highlighted that particular socio-environmental conditions (e.g., low educational level and younger age), psychological processes and attitudes (such as low levels of epistemic trust, the avoidance of uncertainty, extraversion, collective narcissism, and a conspiracy-prone mindset), and contextual factors (e.g., high levels of self-perceived risk and anxiety) seemed to underpin the adherence to beliefs that are not solely the domain of paranoids and extremists but a widespread phenomenon that has caused important health, social, and political consequences during the pandemic. All of this, in the early stages of the pandemic, led to incorrect adherence to virus defense measures (such as wearing masks and social distancing), and now during the vaccination process, it is also (in addition to this) leading to avoidance by part of the population [33,34].

#### 3.3.3. The Health Risks

The health risks related to the infodemic are indirect and direct. Among the indirect risks, there are those related to the non-adherence to defense measures against the contagion and those related to the avoidance of receiving vaccines [45,46]. These lead to the consequent possibility of contracting and spreading the virus. Among the direct consequences, there is adherence to incorrect medical indications. The spread of misinformation is particularly alarming when spread by medical professionals, and existing data on YouTube suggesting vitamin D has immune-boosting abilities can add to viewer confusion or mistrust regarding health information [47]. Further, the suggestions made in the videos may increase the risks of other poor health outcomes such as skin cancer from solar UV radiation [47]. It has been seen that the infodemic has a strong impact on well-being in general [48]. In particular, the scientific literature showed a growth in the direct consequences that had an impact on mental problems. These consequences had an important impact on the psychological sphere, they can even lead to suicide and affect healthcare professionals, patients [35], and common people [36]. In [36], a recent scoping review, articles were analyzed according to average age, gender, and education level; place and period of the studies; exposure time to COVID-19 information; main signs and symptoms related to mental health; main sources of information; suggestions for mitigating the effects of the *infodemic*; knowledge gaps. As a result, it was shown that the most present repercussions of the *infodemic* on adult and elderly mental health were anxiety, depression, and stress, and the most affected groups were young adults and females. A systematic review [35] was performed based on a search from 1 January 2020, to 11 May 2021. Studies that addressed the impact of fake news on patients and healthcare professionals around the world were included. By analyzing the phenomenon of fake news in health, it was possible to observe that *infodemic* knowledge could cause psychological disorders and panic, fear, depression, and fatigue. The extreme consequences of these issues can be represented by suicide as shown in the narrative review in [37]. This narrative review summarized the sociocultural risk and predisposing factors for suicidal behavior in developing countries during the COVID-19 pandemic. The findings revealed fear of being infected, growing economic pressure, and lack of resources due to the lockdown were the factors most responsible in the four countries studied for the current increase in suicides. There were a few cultural differences that were specified in the narrative

#### 3.3.4. Countermeasures

Countermeasures are a very important aspect and must consider the connection with mobile technologies, any involvement in the *health domain*, and whether it is dealing with HLI.

In cases of HLI, the *infodemic* starts directly from scientific works both published after the peer-reviewed process and earlier when they are in the preprint state.

Indeed, the COVID-19 pandemic catalyzed the rapid dissemination of papers and preprints investigating the disease and its associated virus, SARS-CoV-2. We report two initiatives in the case of HLI. In [38], the authors applied a massive online open-publishing approach for improving the publication process using an automatic approach for referenced preprints, journal publications, websites, and clinical trials. Continuous integration workflows retrieved up-to-date data from online sources nightly, regenerating some of the manuscript’s figures and statistics. The proposed architecture improved the interaction of the scientists and minimized the *misinformation*. In [39], the activity by a workgroup (WG) at the Istituto Superiore di Sanità (ISS), the Italian National Institute of Health, was reported. The WG proposed a *workflow* allowing for two cultural mediation activities. A first cultural mediation activity was designed for actors in the *health domain* during the lockdown. During the Italian lockdown, experts in the WG selected scientific works that were constantly reviewed by experts in the field both in the WG and externally. The reviews were made available online in appropriate periodical publications [39]. A second cultural mediation activity concerned stakeholders during the Italian lockdown, to whom daily reports on the critical analysis of the newly published preprints were addressed. The Dear Pandemic project is also aimed [49] in this direction. It is a multidisciplinary, social-media-based science communication project, the mission of which is to educate and empower individuals to successfully navigate the overwhelming amount of information circulating during the pandemic with the two aims: (1) to disseminate trustworthy, comprehensive, and timely scientific content about the pandemic to lay audiences via social media; (2) to promote media literacy and information on hygiene practices, equipping readers to better manage the COVID-19 infodemic within their own networks.

Of course, it is not possible to carry out a complete examination of the possible countermeasures, which are far-reaching and can range from an SMS that arrives on your mobile device, a wall poster, or to a radio message of a testimonial. In line with the purpose of this study and considering the role of mobile technology and social media, we report several relevant actions that emerged from the literature search. The same study [29] that investigated the dissemination of the *infodemic* also proposed a comprehensive list of countermeasures, summarized from different perspectives. The authors emphasized, among the most important countermeasures: risk communication and consumer health information; the use of the mobile technology and social media as a means to reach citizens. The key issue is to offer content that is easy to access via mobile technology and that is displayed in a calibrated and understandable way for common people in a *client–server* manner. Alternatively, it is important to send the same types of information in a distributed way through mobile technology as well as via *ICT pushes* such as through dedicated app messages (e.g., WhatsApp), SMS, tweets, and emails.

In [40], the authors reviewed and provided insight regarding methodologies and the construction of content on health information-seeking behaviors (HISBs). A total of 13 survey tools from eight countries were identified after a review. This review [40], in line with the previous review, highlights how tools (surveys) shared by means of mobile technology, in a *client–server* way, could be useful sensors for the decision makers in healthcare.

The *ICT pushes* particularly need to be tuned to the level of technological literacy. An SMS is more suitable for people with low literacy, who do not use smartphones (but simple mobile phones) such as, for example, the elderly. Other systems are more suitable for people with a higher level of literacy. Subtleties in the composition and the language used of messages sent using *ICT pushes* to reach people have been shown to be very important in affecting behavior and minimizing misinformation [41]. In [41], text messages designed to make vaccination salient and easy to schedule boosted appointments and vaccination rates and minimized avoidance.

The study reported in [42] is very important for the relevance of the *ICT push* method. The study explored how the WHO uses its Twitter profile to inform the population on vaccines against the coronavirus, thus preventing or mitigating misleading or false information both in the media and on social networks. The analysis showed that the WHO is decidedly committed to the use of these tools to disseminate messages that provide the population with accurate and scientific information as well as to combat mis- and disinformation about the SARS-CoV-2 vaccination process. Even the use of artificial intelligence (AI) can be useful in this activity. In [43], it was highlighted how AI-based approaches are useful to improving e-health literacy, including AI-augmented lifelong learning, AI-assisted translation, simplification, summarization, and AI-based content filtering. Furthermore, it exhibited usefulness in combating the *infodemic*, presenting the general advantage of matching the right online health information to the right people.

## 4. Discussion

### 4.1. Considerations Emerging from the Study

The term *infodemic* has particularly resonated over the last two years due to the fact of its association with the COVID-19 pandemic. However, it was not coined recently, but was inherited from previous experiences associated with *infodemiology* and *infoveillance* [15,16,17,18]. The scientific production around this theme exploded over the last two years and was strongly linked both to the COVID-19 pandemic and to mobile technologies and social media, the new vehicles for the transmission of information [4]. The study started from the significant increase in the use of these devices during the pandemic as evidenced by national data [6] and confirmed by international data (Table 2 and Table 3 [8]).

There are *three points of view* reported in this review.

The *first point of view* illustrates how: (a) recent statistical reports [8] highlight the widespread use of social media through mobile technology; (b) the positions of international and relevant bodies on this issue (i.e., WHO, UN, UNICEF, UNDP, UNESCO, UN-AIDS, ITU, UN Global Pulse, IFRC, EUC, and CDC) [9,10,11,12] which, in some cases, emerged through joint initiatives that emphasized the role of social media in the dissemination of the infodemic.

The *second point of view* examined the evolution of scientific production in this field. This started from the first important experiences in this sector [15,16,17,18], well before the pandemic and up to the first important recent initiative on an international consensus [25]. It emphasized the role of the first studies [15,16,17,18], the high production over the last two years [13,14], and the strategic importance of the initiative of international consensus that produced a document with recommendations at the international level [25].

The *third point of view* detailed in-depth the emerging issues related to the infodemic in the recent scientific production arranged into four themes: (a) *dissemination methods*; (b) *social factors*; (c) *health risks*; (d) *countermeasures*.

The role of social media in *dissemination methods* has been reiterated by several studies [29,30,31]. *Social factors* [32,33,34] are important regarding the taking root of the phenomenon that can lead to *health risks*, with not only indirect (for example, due to the avoidance of vaccines [34]) but also direct impacts on mental problems [35,36] and predisposition to suicide [37]. Among the most important *countermeasures,* we found those that combated the infodemic using the same tools that caused its spread such as social media and mobile technology. Studies show the usefulness of calibrated ICT pushes [41] from relevant bodies [42] and client–server solutions at the government level, for example, based on surveys [40].

### 4.2. Boundaries of the Considered Studies and Suggestion for Further Research

The studies reported in the review did not take into consideration scientific productions in languages other than English and how research in this area is evolving also using the preprint databases dedicated to COVID-19. In general, the topic is broad, and the reviews (even systematic) focus on specific aspects while not being able to address them all at the same time. Even our study could not analyze all the implications and problems, which, however, with a three-point approach, tried to provide an integrative review by correlating social media developments at the local and global level [6,8], positions of international bodies [9,10,11,12], consensus initiatives [25], and recent scientific productions by selecting wide-ranging reviews and focused article reviews [26,29,30,31,32,33,34,35,36,37,38,39,40,41,42,43], aiming to provide an overview of the problem. We must also consider that, although with caution in regard to the results, useful indications concerning emerging directions of research in this area also come from the preprints accessible on the websites dedicated to the pandemic. While the reliability of the preprint results is postponed to the end of the peer-review process (approximately 1–3 months), looking at these websites immediately allowed us to gain a perspective on the studies in progress (beyond the final results).

Among the most visited websites of preprints dedicated to the pandemic, we found the website COVID-19 SARS-CoV-2 preprints from medRxiv and bioRxiv [50] and the website Researche Square-SARS-CoV-2 and COVID-19 Preprints [51]. The integration with studies also coming from these preprint sites allowed us to close the study with an even broader perspective by integrating the study conducted so far with previews of the directions of research in this field. Hot topics [52,53] for research development emerged from these databases. A prime example is the role of editors in peer-reviewed journals in controlling the infodemic by avoiding excessive volumes of rapid COVID-19 publications with sometimes hasty peer reviews [52]. Another example [53] relates to how the infodemic has a different behavior based on the languages used. A recent study [53] conducted a comparative study of anti-vaxxers’ aggressive behaviors by analyzing tweets in English and Japanese. They found two common features across these languages. First, anti-vaxxers most actively transmit targeted messages or replies to users with different beliefs. Second, the influential users are more likely to receive the most toxic replies from the anti-vaxxers. However, pro-vaccine sites with a low number of followers are subjected to higher hard replies in English, which ask for special support that differs from the Japanese case. This suggests the need for both language-dependent and -independent countermeasures against infodemic. Other studies, from these databases, confirmed the right direction of the countermeasures identified by the studies taken into consideration based on mobile technologies using, for example, surveys at the national level [54] or the support of categorization processes to protect citizens against fake news [55].

### 4.3. Limitations

This review took into consideration a limited number of sources (Google for the first point of view; PubMed for the other two) and only sources in English. However, it should be noted that (a) an important part of scientific production and direct or related initiatives in this area are in national languages other than English (as we highlighted in the case of the Italian CENSIS report [6]); (b) other databases, such as those of the preprints, could provide in real time interesting indications of the development directions on the research in progress [47,48,52,53].

### 4.4. Prospects

As a narrative review, the study focused on various topics with three points of view to try to provide an overall and unitary vision to the phenomenon comprehending (a) the volume of use of social media (a key element of the infodemic) and the position of international health domain bodies; (b) the rapidity of the evolution of scientific production in this field of life sciences; (c) the key emerging issues.

Future studies could be dedicated to deepening each of the issues addressed. Taking into account the rapid evolution of the topic, it could also be useful to transform this study into a living narrative review with periodic updates. To do this, however, it will be necessary to verify the feasibility of this transformation, which must include periodic updates with appropriate editorial tools and methodologies.

## 5. Conclusions

This review focused on the *infodemic* in the COVID-19 era and addressed *three points of view* to allow for an overall picture of the issue.

The *points of view* focused on:

- The volume of use of social media and the position of international health domain bodies;
- The evolution of scientific production in the life sciences;
- Emerging issues.

The study emphasized the key role of social media as a dissemination tool of the *infodemic* among citizens, documented both by the position of international bodies and by a growing scientific production. The impact on citizens, involved and not involved in the *health domain*, depends on various social factors and involves indirect (e.g., vaccine avoidance) and direct risks, e.g., mental problems and the risk of suicide. The widespread diffusion of social media conveyed by mobile technologies also suggests their use as a countermeasure, calibrated based on the citizen’s level of both technological and health literacy. The sending of correctly informative *ICT pushes* to mobile devices and the provision or collection of information aimed at interventions (through *client–server architectures* accessible from mobile devices, based, for example, on electronic surveys) are proving to be effective and promising countermeasures. This review also suggests as future research directions: (a) the face solutions for minimizing the high-level *infodemic* produced, for example, by scientific publications yet at the stage of the peer review or published with a peer review with bias/distortion; (b) focusing on the impact of the *infodemic* considering its spread in different languages.

## Figures and Tables

**Table 1:** Parameters used for the qualification (* 1.3 was used for studies published in the first three months, and 1.15 was used for studies published in the period ranging from three to six months).

	Score (1 = Minimum; 5 = Maximum)	Weighting
Is the introduction adequate?		N.A.
Is the research design appropriate?		N.A.
Are the methods adequately described?		N.A.
Are the results clearly presented?		N.A.
Are the conclusions supported by the results?		N.A.
Added contribution to the field		*p* = 1.3 or 1.15 *
Topicality level of the review		N.A.

**Table 2:** Volume of use of social media by application.

Application	Main Characteristics	Nation	Producer	Users (Monthly)
WeChat	App for Messaging, mobile payment and social media.	China	Tencent Holdings Limited	1.25 billion
TikTok	Video sharing focused on short-form videos. Free to use.	China	ByteDance	837 millions
Snapchat	App for Photo sharing with video functionalities. Free to use.	US	Snap Inc.	348 millions
Twitter	Mini-blogging based on short messages (tweets).	US	Twitter Inc.	330 millions
YouTube	Social media platform for video sharing. Free to use.	US	Google	More than 2 billions
WhatsApp	Messaging platform that allows users to send text messages, multimedia documents, documents, and GPS. Free to use.	US	Meta	More than 2 billions
Instagram	Social networking and multimedia document sharing site. Free to use.	US	Meta	1 billion
Facebook	Social networking service that allows users to send text messages, multimedia documents, documents, GPS, and other numerous functions (e.g., shopping, real-time videos). Free to use.	US	Meta	2.90 billions

**Table 3:** Volume of use of social media with reference to the Internet and mobile technology.

Access to Internet/Social Media	Number of Users (Billions)
Active internet users	4.66
Active mobile internet users	4.32
Active social media users	4.2
Active mobile social media users	4.15

## Data Availability

Not applicable.

## Abbreviations

List of acronyms of the cited institutional bodies.

Acronym	Description
WHO	World Health Organization
CDC	Centers for Disease Control and Prevention
CENSIS	Centro Studi Investimenti Sociali
EUC	European Commission
UN	United Nations
UNICEF	United Nations International Children’s Emergency Fund
UNDP	United Nations Development Programme
UNESCO	United Nations Educational, Scientific, and Cultural Organization
ITU	International Telecommunication Union
IFRC	International Federation of Red Cross and Red Crescent Societies
UNAIDS	Joint United Nations Programme on HIV/AIDS
